# Novel cuproptosis-related prognostic gene profiles in preeclampsia

**DOI:** 10.1186/s12884-023-06215-y

**Published:** 2024-01-10

**Authors:** Xiaotong Tang, Yi Liu, Yuanyuan Zhang

**Affiliations:** Department of Obstetrics, The First Affiliated Hospital of Nanjing Medical University, Nanjing, Jiangsu China

**Keywords:** Cuproptosis, Preeclampsia, Cell death

## Abstract

**Background:**

Preeclampsia (PE) is a pregnancy-specific disorder with complex pathogenesis. Cuproptosis is a novel identified form of programmed cell death, however, the link between cuproptosis and clinical outcomes in PE is still not fully understood. In this study, we searched for cuproptosis-related genes (CRGs) in the placental tissues of normal and PE patients to clarify the importance of cuproptosis in the development of PE and provide potential predictive indicators for the occurrence of PE.

**Methods:**

Using RNA sequencing data in the GEO database, we conducted functional enrichment analysis of Gene Ontology (GO), Kyoto Encyclopedia of Genes and Genomes (KEGG) and Gene Set Enrichment Analysis (GSEA), supported by linear regression model and operating characteristic curve (ROC) curve analysis, and summarized the role of CRGs in preeclampsia.

**Results:**

A total of 2831 differentially expressed genes related to PE were screened through multiple database analyses. After further intersection with 19 reported CRGs, 5 CRGs have been closely associated with the pathogenesis of PE, including *NFE2L2*, *PDHA1*, *PDHB*, *DLD* and *GLS*. *NFE2L2* was identified as a key central gene. Pearson correlation analysis showed that CRGs could be related to several maternal and fetal outcome factors, including the highest pregnancy blood pressure, placenta weight, umbilical blood flow pulsatility index (PI), and neonatal weight. Linear regression equation revealed that the expression of *NFE2L2* is negatively correlated with the highest pregnancy blood pressure and umbilical blood flow PI but positively correlated with placental weight and neonatal weight. QRT-PCR showed that the expression of these CRGs was significantly lower in placental tissues.

**Conclusions:**

This cuproptosis pattern may be a potential prognostic factor in patients with PE and could provide new insights into disease progression.

**Supplementary Information:**

The online version contains supplementary material available at 10.1186/s12884-023-06215-y.

## Background

Preeclampsia (PE) is a progressive multisystem illness involving widespread endothelial dysfunction and vasospasm. It usually manifests as hypertension and proteinuria after 20 weeks of gestation or postpartum or hypertension and terminal organ dysfunction with or without proteinuria, with an incidence rate of 5–10% [1]. Currently, there is no cure for this pregnancy disorder expect for early termination of pregnancy and rapid delivery of the placenta. Therefore, exploring predictive indicators related to the occurrence of PE is of utmost importance for more effective prediction and treatment of PE and for improving maternal and fetal outcomes.

Copper is one of the metal elements necessary for various biological processes, including making energy, connective tissues, and blood vessels [2, 3]. The fluctuation of copper levels in cells has been associated with the occurrence and development of diseases, including tumor cell proliferation, angiogenesis, and metastasis [4–8]. Several copper-regulating ions have been used in anti-cancer treatment, including copper ion carriers (disulfiram, dithiocarbamate, elesclomol.) and copper chelators (tributyl ether, tetrathiomolybdate.) [9, 10].

Cuproptosis is a copper-dependent and regulated non-apoptotic death mode that participates in the occurrence and development of many diseases. Unlike apoptosis, scorch death, necrotic apoptosis, and iron-related death, cuproptosis occurs through the direct combination of copper and the fatty acylation component of the tricarboxylic acid cycle (TCA) [11, 12], which leads to the aggregation of fatty acylated proteins, the loss of iron-sulfur cluster proteins, and protein toxicity stress and cell death. Recent studies have identified several genes related to cuproptosis, which may provide a new strategy for predicting the prognosis of patients with PE [13, 14].

It was known that cuproptosis was involved in tumor cells’ metabolic programs, such as hepatocellular cancer, lung cancer [15–17]. Besides, cuproptosis-related genes (CRGs) has been verified to regulate the migration and invasion of cells by mediating cell death. Nowadays, the mechanism of PE progression has not been clarified, and obstruction of uterine spiral artery remodeling is the most convincing hypothesis. In this hypothesis, the weakness of trophoblasts’ invasiveness is the key to explain why uterine spiral artery remodeling obstructed.

In the present study, we aimed to comprehensively clarify the molecular alterations and clinical relevance of cuproptosis in the development of PE and verified the role of CRGs in the PE progression by validating common gene profiles in multiple databases and clinical models. Based on the above, we intended to provide potential predictive indicators for the occurrence of PE and lay a foundation for the therapeutic application of cuproptosis regulators in PE.

## Methods

### Multi-omics data source and preprocessing

RNA sequencing dataset (GSE75010) and corresponding clinical data of patients with PE and non-PE were downloaded from the GEO database (https://www.ncbi.nlm.nih.gov/geo). This dataset contains gene expression data of 77 normal placental tissues and 80 PE placental tissues. The genes related to cuproptosis were derived from cuproptosis articles. In that article, whole genome CRIPSR/Cas9 positive selection screen using two copper ionophores (Cu-DDC and elesclomol-copper) in OVISE cells. Overlapping hits with FDR score < 0.01 were analyzed [18]. The R language “limma” software package was used to analyze the gene expression difference between PE patients and normal tissues. In order to obtain more DEGs, | LogFC > 0.1 | and *p* < 0.05 were set as thresholds. The visualization of DEG was achieved by building volcano maps, heat maps. Later, the CRGs were used to intersect with DEGs and obtain the DEGs related to cuproptosis between PE patients and normal pregnant women. Also, the differences in expression between healthy pregnant women and PE pregnant women were analyzed.

### Gene network and gene enrichment analysis

Gene changes at the genomic level can be identified using microarray technology and bioinformatics analysis. In recent years, bioinformatics methods have been widely used in addition to various other analyses to analyze microarray data to identify DEGs [19–21]. The central genes were identified by protein-protein interaction (PPI) network analysis and ten algorithms of the cytoHubba plug-in. In addition, Gene Ontology (GO), Kyoto Encyclopedia of Genes and Genomes (KEGG) [16, 22–24], and Gene Set Enrichment Analysis (GSEA) were used to determine the potential functions of biomarkers.

### Weighted gene co-expression network analysis (WGCNA)

The R language “WGCNA” software package was used to construct a gene co-expression network. First, after clustering the samples according to clinical information, an outlier threshold of 60 was set to screen out outliers. Next, based on the soft threshold parameter β, power function f (x) = x β was used to Convert the Pearson correlation matrix to a weighted adjacency matrix. The appropriate soft threshold was determined. After selecting the power of 6, the weighted adjacency matrix was transformed into a topological overlap matrix (TOM) with topological overlap (TO) based dissimilarity (1-TOM), and clustering was performed through TOM. Finally, genes with similar expression patterns were classified into modules according to the difference in TOM of each gene for average linkage hierarchical clustering. To further analyze each module, the minimum size (genome) of the gene tree was 30. If the distance was < 0.25, the modules were merged. After obtaining co-expressed gene modules, central genes were screened according to the criteria of gene weight (GS) > 0.2, GS *p* value < 0.05, and module weight (MM) > 0.8 MM *p* value < 0.05.

### Criterion and tissue collection

We collected general information from 40 pregnant women who undergoing obstetric examination at the First Affiliated Hospital of Nanjing Medical University from January 2021 to January 2022 based on inclusion criteria. Inclusion criteria: singleton pregnancy, no hypertension and diabetes before pregnancy, regular prenatal examination, no fetal abnormalities. Then, 40 pregnant women were divided into control group and PE group based on the PE definition of guidelines related to gestational hypertension published by AOCG in 2020 [25].

Placenta tissues was taken during cesarean section with the patient’s consent before surgery. The placental tissue samples were obtained from the midsection between the chorionic and maternal basal surfaces at different placenta locations, and each sample measured approximately 1 cm × 1 cm × 1 cm. The samples were then washed in PBS buffer and rapidly frozen in liquid nitrogen. Written informed consent was obtained from all patients. This study was approved by the Ethics Committee of the First Affiliated Hospital of Nanjing Medical University (registration number: 2018-SR-252).

### Real-time fluorescence quantitative (PCR)

Total RNAs were isolated from the placenta or cells in accordance with the standard TRIzol protocol (Life Technologies). RNA (1 μg) was used for cDNA synthesis by using the HiScript III RT SuperMix for qPCR (Vazyme, Nanjing, China) in accordance with the manufacturer’s instructions. qPCR procedure was carried out by using ChamQ SYBR qPCR Master Mix kit (Vazyme, Nanjing, China) in accordance with the manufacturer’s instructions. The abundance of mRNA was quantified using the 2 − ΔΔCT method, which was normalized to the expression level of GAPDH and converted to fold changes, qRT-PCR was performed as previously described [26].

### Statistical analysis

First, we made a descriptive statistical analysis of the PE patients in the database. Shapiro Wilk test was used to determine whether continuous data had a normal distribution. If the data conformed to the normal distribution, a T-test was used to compare the general situation of normal and PE patients and the maternal and fetal outcomes, expressed by mean ± standard deviation. If the data did not conform to the normal distribution, the Kruskal Wallis rank sum test was used to compare the differences between the two groups, expressed by the median value [Q1 quartile - Q3 quartile]. The categorical variables are expressed by frequency and proportion (n [%]). The Chi-square test was used to compare the differences between groups. Benjamin-Hochberg’s method was used to make multiple corrections. FDR < 0.05 indicated a statistically significant standard. Through database analysis, we used linear regression to correlate CRGs with maternal and fetal outcomes of PE. The predictability of biomarkers was analyzed by the receiver operating characteristic (ROC) curve. The *p*-value was bilateral, and *p* < 0.05 indicated statistical significance.

## Results

### DEGs and CRGs in the placenta of PE

First, differences in maternal age, body mass index (BMI), peak blood pressure (systolic/diastolic blood pressure), termination week, umbilical blood flow S/D ratio, Apgar score (1 min, 5 min), were assessed between patients (PE pregnant women) and controls (normal pregnant women) (Table 1). Pregnancy termination in PE patients occurred more than one week earlier compared to normal pregnant women (*p* = 0.013), and the BMI and peak blood pressure (systolic and diastolic blood pressure) in this group were significantly higher than in healthy pregnant women (*p* = 0.0264; *p* = 0.000; *p* = 0.000), while the 1-minute Apgar score of newborns in PE pregnant women was significantly lower (*p* = 0.0432). The two groups had no significant difference in other factors (all *p* > 0.05).

**Table 1:** Comparison of general conditions of pregnant women between the two groups

	Normal (N = 77)	PE (n = 80)	*P*-value	Statistic
Maternal age (Year)	33.2 (5.35)	33.2 (5.89)	0.995	-0.0063
Maternal BMI (kg/m^2^)	24.5 (4.84)	26.5 (5.64)	**0.0264**	-2.2448
Gestational week (Week)	34.0 (4.73)	32.3 (3.57)	**0.013**	2.5149
Umbilical cord diameter	1.21 (0.290)	1.16 (0.393)	0.38	0.8809
Mean umbilical PI	1.30 (0.463)]	1.39 (0.379)	0.319	-1.0046
Maximum systolic BP	136 (23.5)	170 (18.1)	**< 0.001**	-10.0190
Maximum diastolic BP	85.3 (14.7)	107 (9.73)	**< 0.001**	-11.0239
Apgar .score 1 min	7.93 (1.82)	7.30 (1.76)	**0.0432**	2.0409
Apgar score 5 min	8.70 (1.06)	8.71 (0.725)	0.935	-0.0821

Note: Shapiro Wilk test determines whether continuous data had a normal distribution. Measurement data using Independent-sample t test, normal distriution of data to mean(standard deviation, SD). Abbreviations: preeclampsia (PE); body mass index (BMI); pulsatility index (PI); blood pressure (BP)

**Fig. 1:**
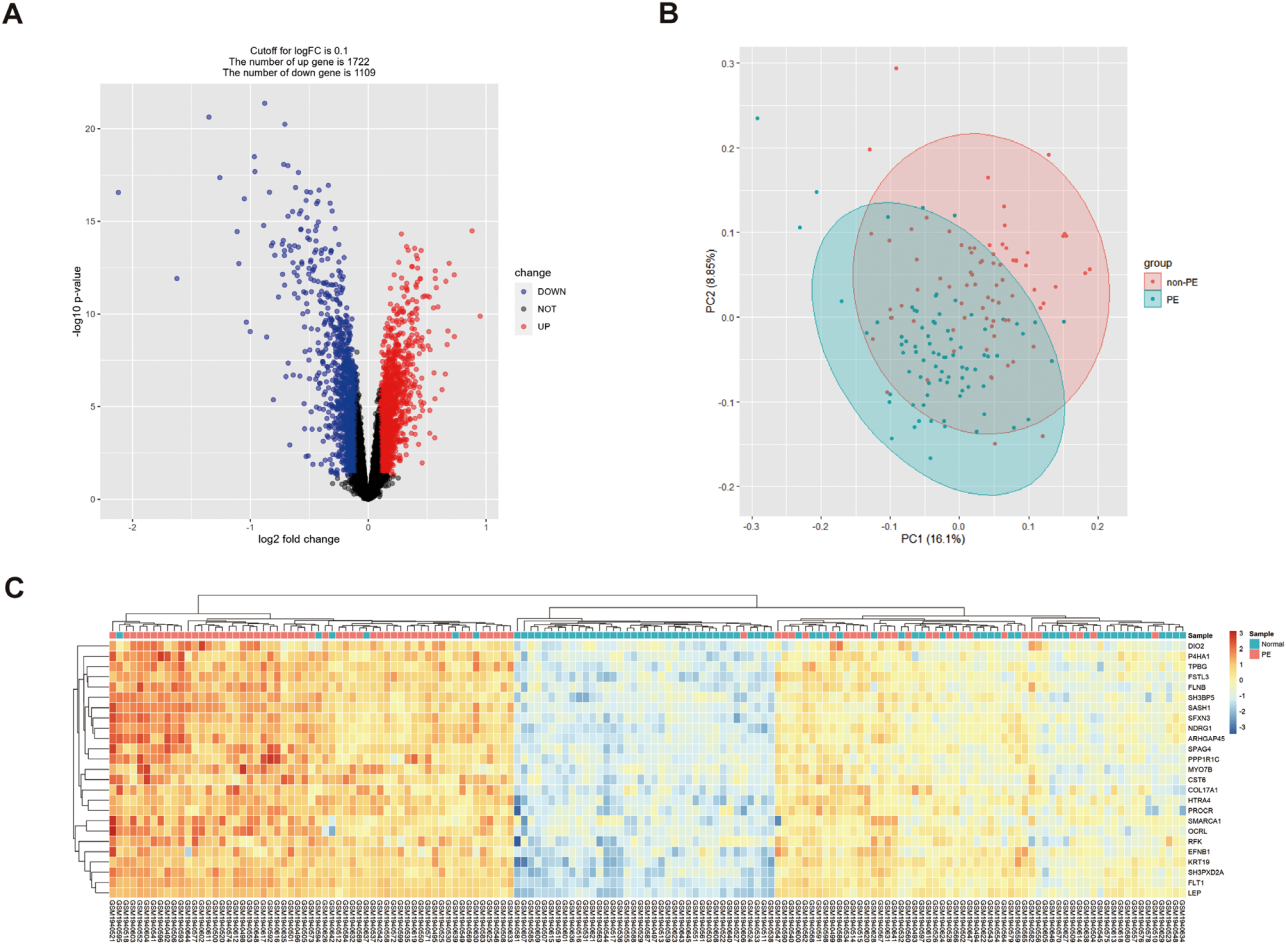
DEGs in normal placenta tissue and PE placenta tissue. (**A**) Volcanic map shows DEGs in between the PE placenta tissues and the normal placenta tissues. Blue represents normal placental tissue and red represents preeclampsia placental tissue (logFC > 1 and *p*-value < 0.05). (**B**) Principal component analysis shows 1722 upregulated genes and 1109 downregulated genes involved in the PE compared to non-PE in GSE75010 datasets. (**C**) Heatmap shows differential expression of the top 25 genes among 2831 PE risk genes among a number of samples

After data standardization and differential gene expression analysis, 2831 DEGs were found in normal placental tissue and PE placental tissue, including 1722 upregulated and 1109 downregulated genes (Fig. 1A-B), compared with PE placental tissue; partial DEG expression was assessed using a corrected *p*-value through a heat map (Fig. 1C).

### Multiple analysis of GO, KEGG, and GSEA

**Fig. 2:**
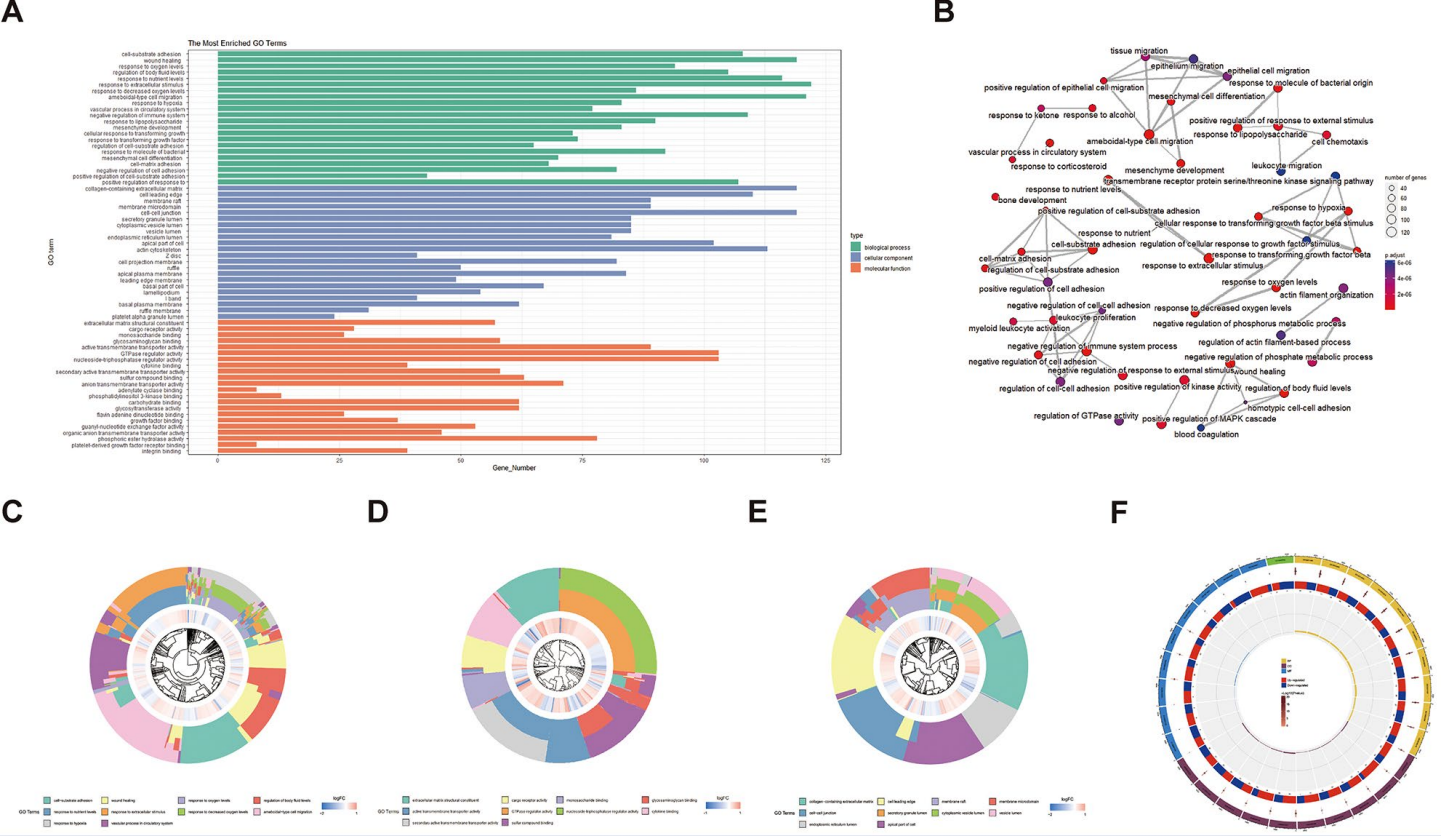
GO analysis. (**A**) GO functional enrichment analysis results for DEGs. (**B**) PPI network of DEGs. The circle size represents the number of genes involved in this pathway, and the smaller the *p*-value, the bluer the color. (**C-E**) Go analysis: clustering diagram of BP, CC, MF enrichment system. The first circle shows the first 10 biological processes in GO enrichment analysis, and the second circle shows the correlation between DEGs and BP/CC/MF. (**F**) The circle diagram was formed by integrating and enriching the results of the circle R package. BP biological process; CC cellular component; MF molecular function; |log2(fold change)| > 2/3 and *p* < 0.05

**Fig. 3:**
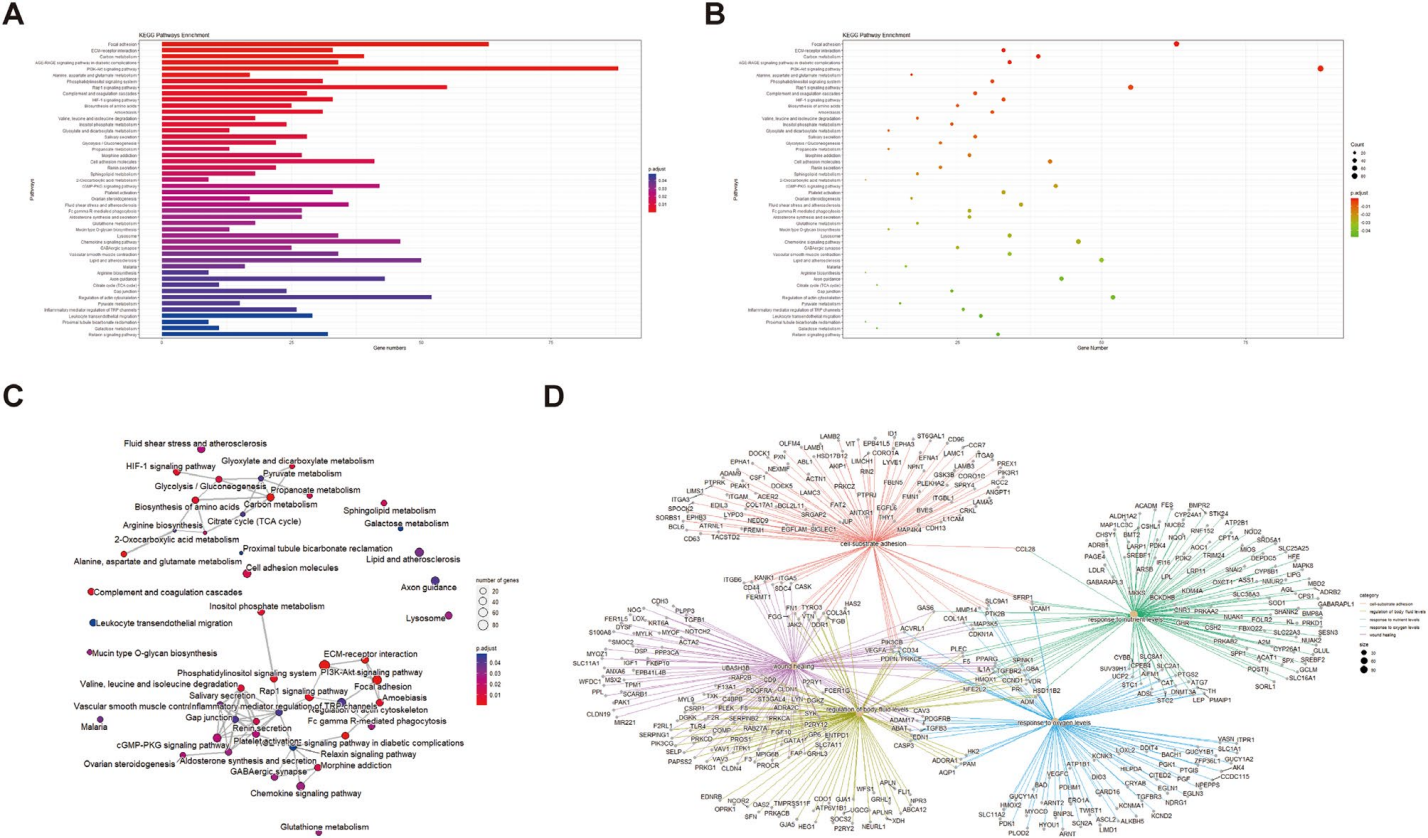
KEGG analysis. (**A**) The first 50 pathways of KEGG enrichment analysis. Different column lengths in the figure represent the number of genes in the KEGG secondary classification. The longer the column, the more genes in the classification. (**B**) Bubble diagram of the first 50 pathways enriched by the KEGG pathway. The size of the dot indicates the number of genes. The larger the dot, the more genes enriched in the pathway; the redder the dot, the smaller the *p-*value, indicating the more significant the pathway. (**C, D**) KEGG Enrichment Advanced Network Diagram displays the connection information between each pathway and the information of potential key genes in the pathway. The node size represents the number of genes enriched to this pathway after KEGG enrichment analysis, the gradient color of the node represents the *p-*value of KEGG enrichment analysis, and the gray node shows the adjacent pathway supplemented from the KEGG connection database

**Fig. 4:**
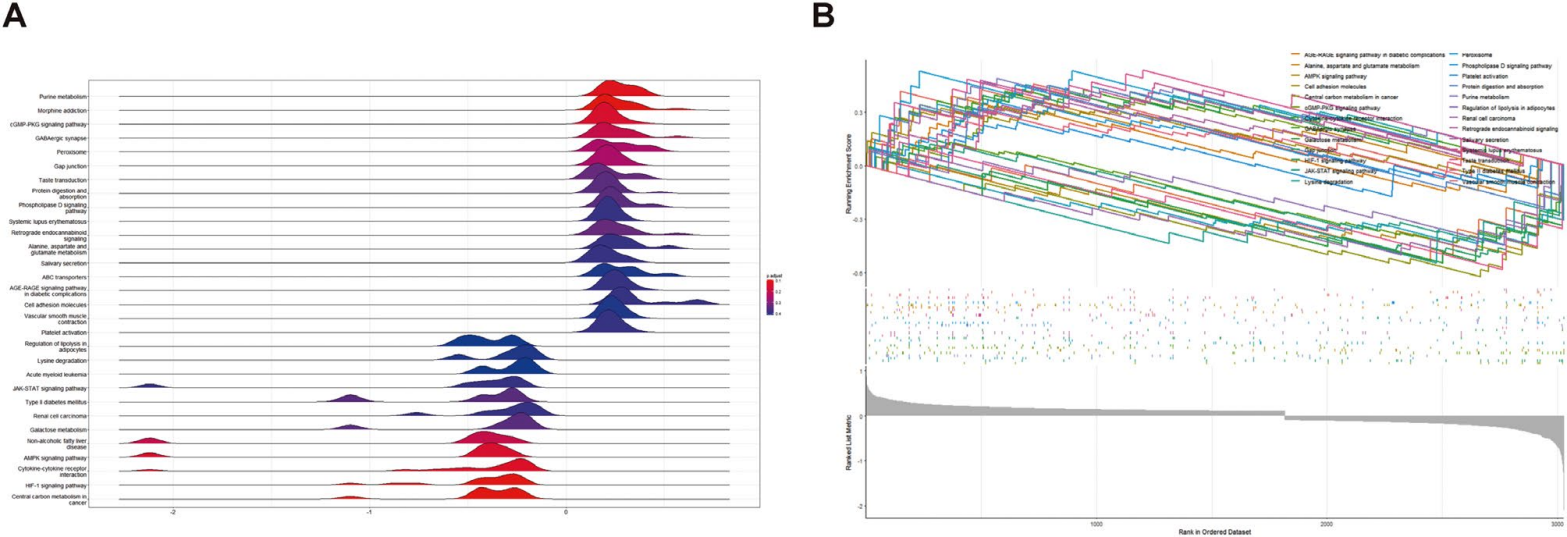
GSEA analysis. (**A**) GSEA enrichment map: upregulation or downregulation of all channels. The redder the color, the smaller the *P* value. (**B**) Enrichment analysis diagram of the first 30 enrichment pathways screened based on *p-*value: the enrichment scores of all genes in this pathway are connected into a line, and the peak is the enrichment score (ES). The gray map below the line shows the rank value distribution of all genes. The ordinate is the rank list metric, which can be understood as the FC value after formula processing

In order to understand the biological processes involved in these DEGs, GO, KEGG, and GSEA analyses were performed. The GO pathway revealed that DEGs are mainly enriched in oxidative stress, hypoxia reaction, intercellular connection, and GTPase (Fig. 2A-F). KEGG pathway found that DEGs were mainly enriched in the PI3K pathway and atherosclerosis (Fig. 3A-D). After genome analysis by the GSEA pathway, it was found that DEGs were mainly concentrated in purine metabolism, cGMP PKG signal pathway, GABA synapse, and other pathways (Fig. 4A-B).

### WGCNA analysis of cuproptosis genes associated with PE

**Fig. 5:**
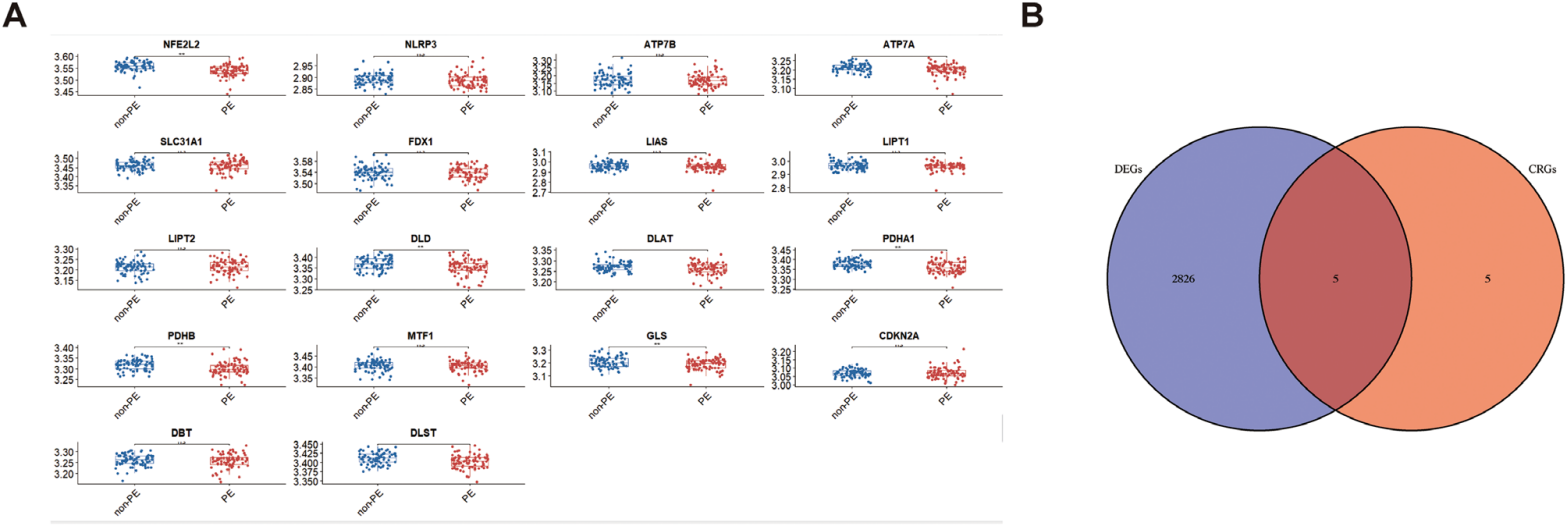
Differential expression and genetic changes of CRGs in PE. (**A**) Scatter plot. The expression of 19 CRGs in PE placenta tissues and normal placenta tissues (red in PE and blue in normal). The upper and lower ends of the box represent the quartile range of values, while the line in the box represents the median value. *p < 0.05, **p < 0.01. (**B**) Venn diagram. There are five common genes in the transcriptome database set of DEGs and CRGs data sets, including “NFE2L2”, “PDHA1”, “PDHB”, “DLD” and “GLS”

**Fig. 6:**
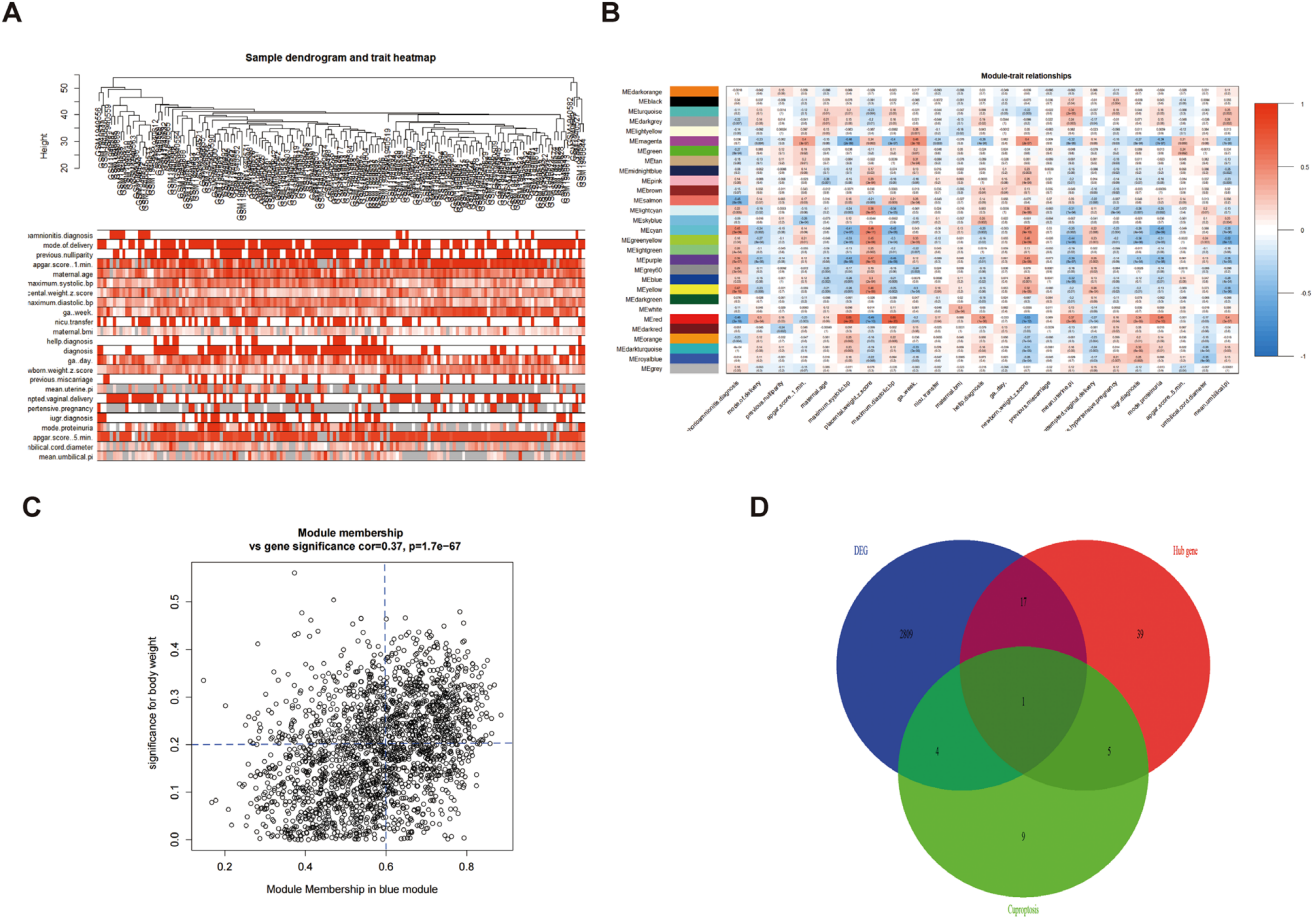
WGCNA analysis. (**A-B**) Based on the weighted correlation, hierarchical clustering analysis is conducted, and the clustering results are segmented according to the set criteria to obtain different gene modules represented by the branches and different colors of the clustering tree. The vertical axis is the gene module and the horizontal axis is the clinical representation. Red represents a positive correlation. Blue represents a negative correlation. (**C**) Gene scatter map in blue gene module: Selecting interested driver genes from key models. Hub gene screening criteria: MM > 0.6, GS > 0.2; (**D**) Venne diagram of DEG, CRG, Hub gene: *NFE2L2* is the key gene

First, we statistically analyzed the differences in the expression of 19 cuproptosis genes in normal placenta tissues and PE placenta tissues in the database. *NFE2L2, PDHA1, PDHB, DLD*, and *GLS* genes significantly differed between the two groups (Fig. 5A), and the five differential genes were downregulated in PE (Table 2; Fig. 5B). According to genes with a similar expression, DEGs were grouped into modules using WGCNA, and 28 modules were finally identified (Fig. 6A). Then, Pearson correlation coefficient analysis was conducted to connect each module with the clinical characteristics of pregnant women (including systolic blood pressure, diastolic blood pressure, umbilical artery blood flow ratio) and maternal and fetal outcomes (neonatal APGAR score, neonatal weight, placental weight). The results showed that the negative correlation coefficient of the blue module was high, indicating that it has a protective role in the occurrence and development of PE (Fig. 6B). According to the cut-off standard (| MM | > 0.8 and GS > 0.2), 62 genes in this module were identified as high-weight central genes. Finally, the Wayne map was constructed to intersect DEGs, CRGs, and hub genes. The intersection of DEGs, hub genes, and CRGs from GSE75010 obtained a common central gene: NFE2L2 (Fig. 6C-D).

**Table 2:** Intersection of differential gene and copper death gene

Gene	logFC	AveExpr	T	*P*.Value	adj.*P*.Val	β	change
NFE2L2	0.16	10.70	5.49	1.58E-07	5.43E-06^*^	6.98	UP
PDHA1	0.13	9.34	4.49	1 38E-05	2 29E-04^*^	2.72	UP
PDHB	0.11	8.92	3.69	3.06E-04	2.87E-03^*^	-0.19	UP
DLD	0.12	9.27	3.28	1.27E-03	8.67E-03^*^	-1.50	UP
GLS	0.11	8.19	2.55	1 17E-02	4 47E-02^*^	-3.50	UP

*p < 0.05

### Pearson correlation coefficient analysis of cuproptosis genes associated with PE

**Fig. 7:**
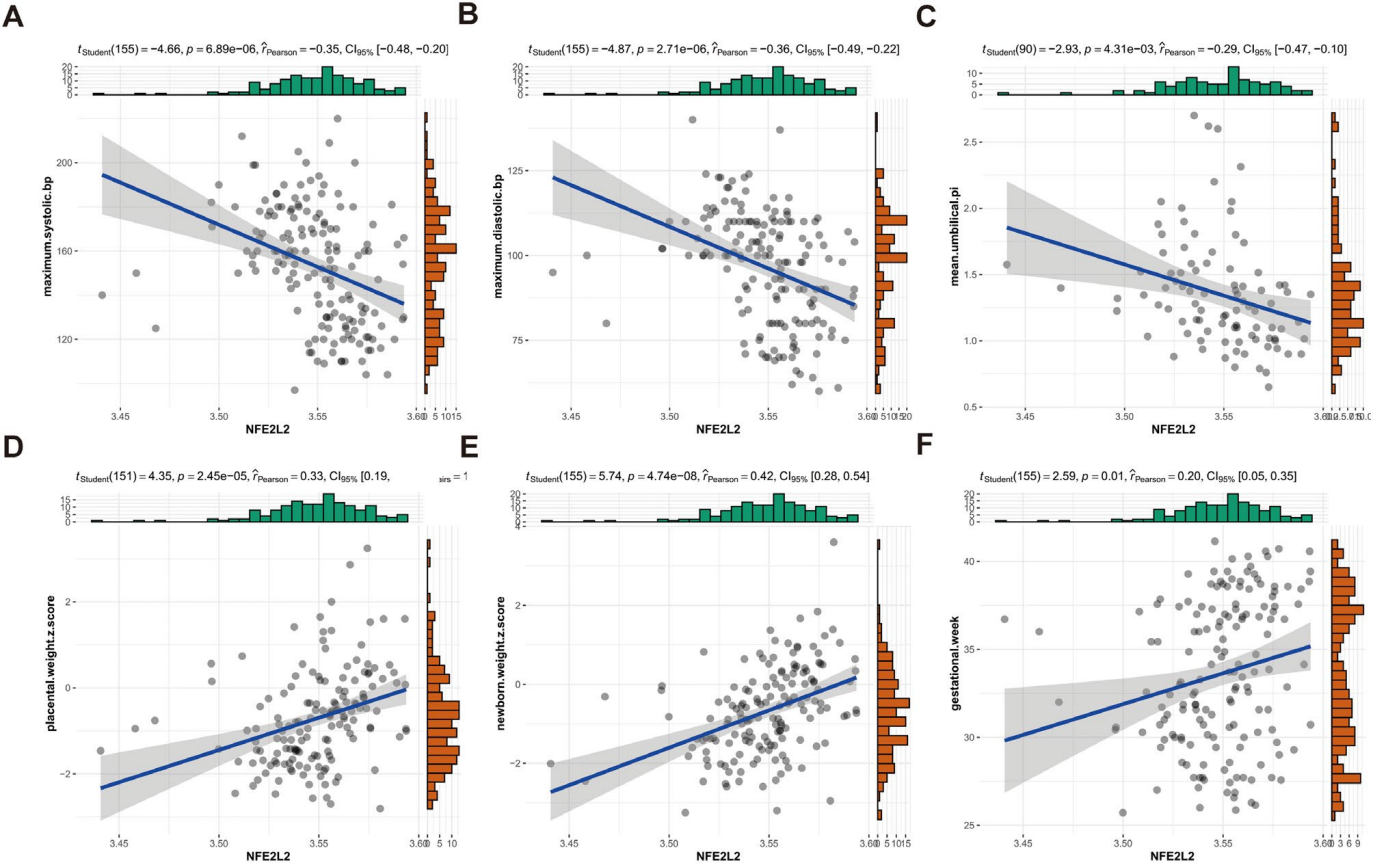
Linear regression model. (**A-F**) A linear relationship between *NFE2L2* gene expression and pregnancy systolic blood pressure, diastolic blood pressure, umbilical artery blood flow ratio, placental weight percentile, and fetal weight percentile

In order to further study the relationship between the central gene and maternal blood pressure and related factors of maternal and fetal outcomes, Pearson correlation analysis was used, showing that “NFE2L2”, “PDHA1”, “PDHB”, “DLD”, “GLS” were associated with peak blood pressure (systolic and diastolic), termination of pregnancy, umbilical blood flow PI, placental weight and neonatal weight. The results showed that the expression of “NFE2L2” was negatively correlated with the peak blood pressure (systolic and diastolic blood pressure) [log2(fold change) = -7.87, *p* = 6.89e-06; log2(fold change) = -8.75, *p* = 2.71e-06], umbilical blood flow PI [log2(fold change) = -2.14, *p* = 4.31e-03](Fig. 7A-C), but positively correlated with placental weight [log2(fold change) = -6.68, *p* = 2.45e-05], placental weight [log2(fold change) = -2.14, *p* = 4.31e-03] and neonatal weight [log2(fold change) = -12.59, *p* = 4.74e-08](Fig. 7D-F). Other genes were related to some maternal and fetal outcomes, such as “PDHA1” and “DLD”, which were inversely proportional to peak blood pressure (systolic and diastolic blood pressure) and were proportional to placental weight and neonatal weight. However, there was no correlation between the termination week of pregnancy and umbilical blood flow PI (Figure S1-S2). “PDHB” was negatively correlated with peak blood pressure (systolic and diastolic blood pressure) and umbilical blood flow PI, while it was positively correlated with placental weight and neonatal weight; however, it did not affect the termination of pregnancy (Figure S3). “GLS” was negatively correlated with peak systolic blood pressure and umbilical artery PI and positively correlated with placental weight, while there was no other correlations (Figure S4).

### ROC curve analysis of the potential of the cuproptosis gene as a predictor of PE

**Fig. 8:**
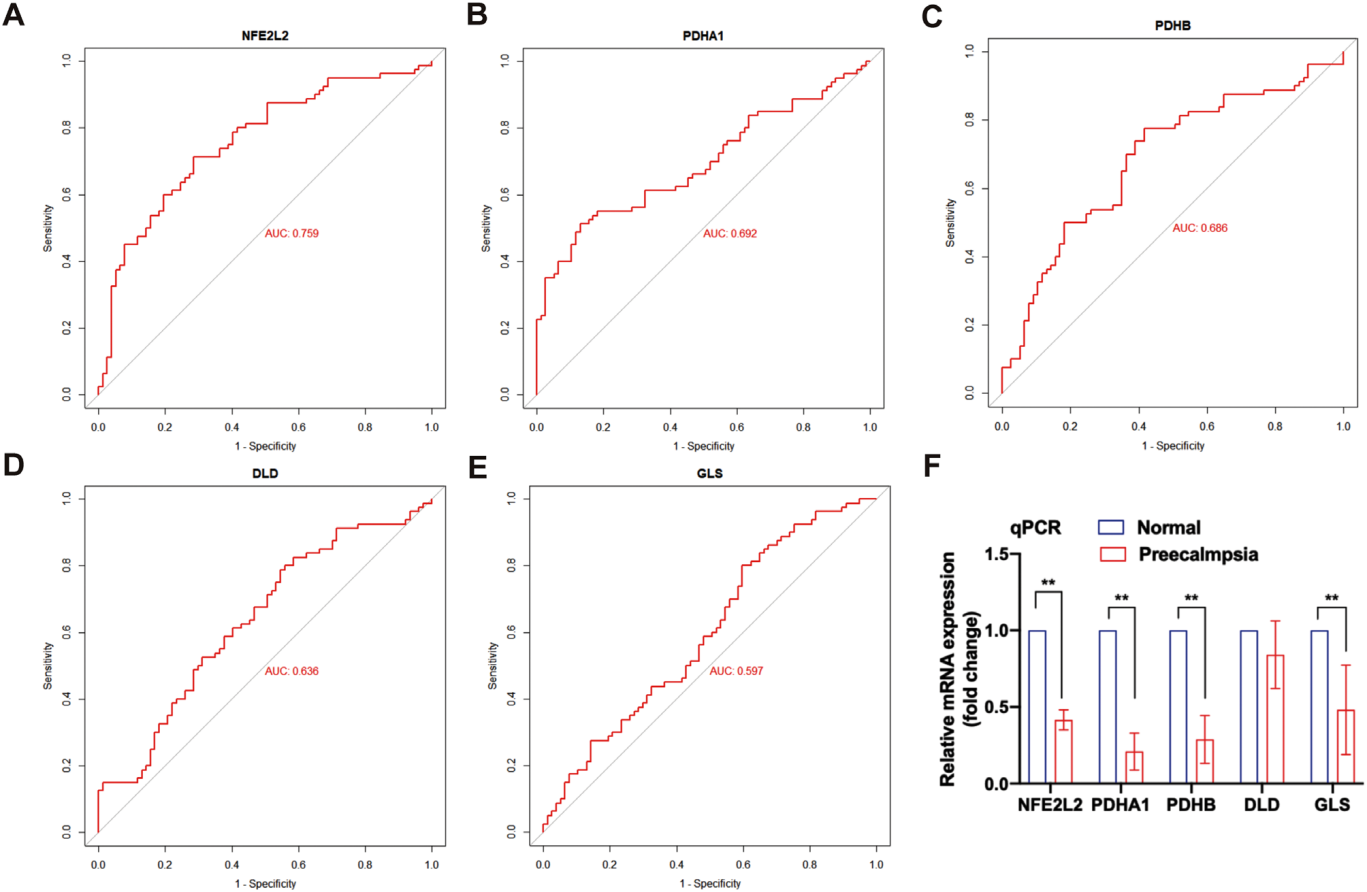
Receiver operating characteristic curve (ROC) and qRT-PCR. (**A-E**) ROC curve of *NFE2L2, PDHA1, PDHB, DLD*, and *GLS* in pregnant women diagnosed with PE; **F**. qRT-PCR was used to verify the difference of *NFE2L2, PDHA1, PDHB, DLD*, and *GLS* expression among 6 pairs of placental tissues

Based on the correlation between the above factors related to maternal and fetal outcomes and the cuproptosis genes, the results showed that the area under the ROC curve (AUC) values of *NFE2L2, PDHA1, PDHB, DLD*, and *GLS* were 0.759, 0.692, 0.686, 0.636, and 0.597, respectively, regarding their potential to distinguish the markers between PE patients and normal pregnant women (Fig. 8A-E). These results pointed out that *NFE2L2* is the most closely related cuproptosis gene that can predict the occurrence and development of PE, followed by *PDHA1, PDHB*, and *DLD.* GLS resulted as the least closely related to PE. To further clarify the potential influence of the above CRGs on the occurrence of PE, we conducted a qRT-PCR experiment, which showed that in addition to “GLS”, the other four genes had significantly low expression in PE placenta, with a statistically significant difference (*p* < 0.05)(Fig. 8F).

## Discussion

In the present study, we first analyzed the general situation of normal pregnant women and PE pregnant women in GSE data, finding that the BMI, systolic blood pressure, and diastolic blood pressure were significantly higher in PE pregnant women. Also, the termination of pregnancy occurred earlier in patients with PE compared to healthy pregnant women. On this basis, 2831 DEGs between normal placental tissue and PE placental tissue were screened in the database, including 1722 upregulated and 1109 downregulated genes. Combined with 19 genes related to cuproptosis in the current cancer database, *NFE2L2, PDHA1, PDHB, DLD*, and *GLS* showed significant differences between the two groups. Therefore, we speculated that cuproptosis has an important role in regulating the occurrence of PE, indicating the potential role of cuproptosis in predicting PE.

Through hierarchical clustering, adjacency relationship, and heat map analysis, we found that *NFE2L2, PDHA1, PDHB, DLD*, and *GLS* have potential roles in predicting and diagnosing PE. In order to clarify that these genes can potentially affect the maternal and fetal outcomes of PE, we assessed the association between these genes and maximum systolic blood pressure, maximum diastolic blood pressure, umbilical artery PI ratio, uterine artery PI ratio, the last week of pregnancy, and neonatal weight of pregnant women. Our results showed that *NFE2L2* is the cuproptosis gene most closely related to the occurrence of PE, and its upregulation could significantly improve maternal and fetal outcomes. The other four cuproptosis genes were related to some maternal and fetal outcomes, suggesting that the increased expression of *PDHA1, PDHB, DLD*, and *GLS* can improve maternal and fetal prognosis to varying degrees.

Next, ROC curve analysis was used to determine whether the fluctuations in the expression of CRGs could affect the occurrence and development of PE. In order to further determine whether the above four cuproptosis genes could be used as predictive markers of PE for in-depth research, we used qRT-PCR, finding that *NFE2L2, PDHA1, PDHB*, and *DLD*, as screened CRGs, could affect the progress of PE by regulating cuproptosis-related mechanisms. However, *GLS* had no significant difference in the expression of PE placental tissue.

Many studies have proved that cuproptosis mediates cell death by regulating the TCA cycle. In this study, four cuproptosis genes (i.e., *NFE2L2, PDHA1, PDHB*, and *DLD*) were found to have different roles in cuproptosis [27, 28]. The transcription factor encoded by *NFE2L2* - nuclear factor red cell 2 related factor 2 (NRF2) is a key regulator of antioxidants, which maintains homeostasis by detecting the level of oxidative stress in cells. In its inactive form, NRF2 combines with its negative regulator KEAP1. Yet, when reactive oxygen species (ROS) appear, the cysteine residue of KEAP1 is modified, and KEAP2 is separated from NRF2. Then, NRF2 is transferred to the nucleus and acts as a transcription factor in combination with antioxidant response elements. NRF2 is currently recognized as a potential tumor therapeutic target [29, 30].

*PDHA1* and *PDHB* are part of pyruvate dehydrogenase (PDH) complexes, a nuclear-encoded mitochondrial multienzyme complex that catalyzes the overall conversion of pyruvate to acetyl coenzyme a and CO2 and provides the main link between glycolysis and the TCA cycle. The PDH complex is composed of multiple copies of three enzyme components: pyruvate dehydrogenase (E1), dihydroceramide acetyltransferase (E2), and thiamine dehydrogenase (E3). *PDHA1* encodes E1 containing E1 active site α and has a crucial role in the function of the PDH complex. PDHB encodes E1 β Yaki [31, 32]. Dihydrothiamide dehydrogenase (DLD) encodes a class I pyridine nucleotide disulfide oxidoreductase family member.

As a mitochondrial protein, *DLD* has an important role in the energy metabolism of eukaryotes; it participates in at least five multi-enzyme complexes and is a necessary component for the complex to complete the reaction. In addition, *DLD*, as a flavoprotein oxidoreductase, uses FAD as a cofactor to receive protons and electrons to catalyze the formation of disulfide bonds [33, 34]. *PDHA1*, *PDHB* and *DLD* have an impact on the mitochondrial tricarboxylic acid cycle.

## Strengths and limitations

The present study has multiple strengths. First, this is the first study that explored the role of cuproptosis in the occurrence and development of PE. Cell death has always been a focus of interest in cancer research. Many studies have proved that cell death is the basis of tumor occurrence and development [24, 35–37]. Cuproptosis is a new type of cell death, which is different from other cell death mechanisms and mainly depends on mitochondrial respiration. In this study, we screened and compared the database and verified it in placental tissue using qRT-PCR technology. It was found that NFE2L2, PDHA1, PDHB, and DLD, as selected prognostic genes, can affect the progress of PE by regulating cuproptosis-related mechanisms. In addition, the currently recognized hypothesis suggests that the occurrence and development of PE is caused by disorders in uterine spiral artery remodeling. The most important factor among them is usually due to the decreased invasion and migration ability of trophoblasts, so it is crucial to verify the changes in the expression levels of related genes in placenta tissues before further research on the mechanism of cuproptosis can be conducted. In order to further explore the role of copper death related genes in the mechanism of PE and whether it can be used as a serum marker to predict the occurrence of PE is our future research direction.

The present study also has some limitations. First, although these genes were screened through the database and the validation of clinical tissue samples, the number of tissue samples for validation was not large enough. Secondly, the specific regulatory molecular mechanisms of CRG in PE need to be further investigated.

## Conclusions

In this study, the related genes that can regulate the progress of PE were analyzed by combining the database data, along with the reported CRGs, and concluded that *NFE2L2, PDHA1, PDHB*, and *DLD* might have a crucial role in PE. These data enhanced our knowledge of molecular mechanisms underlying of PE, and may provide a new direction for developing therapeutic strategies related to cuproptosis for PE prevention and treatment.

### Electronic supplementary material

Below is the link to the electronic supplementary material.

**Supplementary Material 1:** The raw material of validation of differential expression of copper death genes in human placental tissue using RT qPCR technology

**Supplementary Material 2: Figure S1** Linear regression model. (A-F). Linear relationship between PDHA1 gene expression and pregnancy systolic blood pressure, diastolic blood pressure, umbilical artery blood flow ratio, placental weight percentile, fetal weight percentile. **Figure S2** Linear regression model. (A-F). Linear relationship between PDHB gene expression and pregnancy systolic blood pressure, diastolic blood pressure, umbilical artery blood flow ratio, placental weight percentile, fetal weight percentile. **Figure S3** Linear regression model. (A-F). Linear relationship between DLD gene expression and pregnancy systolic blood pressure, diastolic blood pressure, umbilical artery blood flow ratio, placental weight percentile, fetal weight percentile. **Figure S4** Linear regression model. (A-F). Linear relationship between GLS gene expression and pregnancy systolic blood pressure, diastolic blood pressure, umbilical artery blood flow ratio, placental weight percentile, fetal weight percentile

## Data Availability

All data generated or analyzed during this study are included in this published article. https://www.ncbi.nlm.nih.gov/geo. along with the accession number (GSE75010).
