# Supplementary material for: Novel cuproptosis-related prognostic gene profiles in preeclampsia
Source: BMC Pregnancy Childbirth. 2024 Jan 10;24:53. doi: 10.1186/s12884-023-06215-y (PMC10777556; doi:10.1186/s12884-023-06215-y)
Supplement: Supplementary file 2 — Supplementary Material 2: Figure S1 Linear regression model. (A-F). Linear relationship between PDHA1 gene expression and pregnancy systolic blood pressure, diastolic blood pressure, umbilical artery blood flow ratio, placental weight percentile, fetal weight percentile. Figure S2 Linear regression model. (A-F). Linear relationship between PDHB gene expression and pregnancy systolic blood pressure, diastolic blood pressure, umbilical artery blood flow ratio, placental weight percentile, fetal weight percentile. Figure S3 Linear regression model. (A-F). Linear relationship between DLD gene expression and pregnancy systolic blood pressure, diastolic blood pressure, umbilical artery blood flow ratio, placental weight percentile, fetal weight percentile. Figure S4 Linear regression model. (A-F). Linear relationship between GLS gene expression and pregnancy systolic blood pressure, diastolic blood pressure, umbilical artery blood flow ratio, placental weight percentile, fetal weight percentile [file 12884_2023_6215_MOESM2_ESM.docx]

**Figure S1 Linear regression model.** **(A-F).** Linear relationship between PDHA1 gene expression and pregnancy systolic blood pressure, diastolic blood pressure, umbilical artery blood flow ratio, placental weight percentile, fetal weight percentile.

**Figure S2 Linear regression model.** **(A-F).** Linear relationship between PDHB gene expression and pregnancy systolic blood pressure, diastolic blood pressure, umbilical artery blood flow ratio, placental weight percentile, fetal weight percentile.


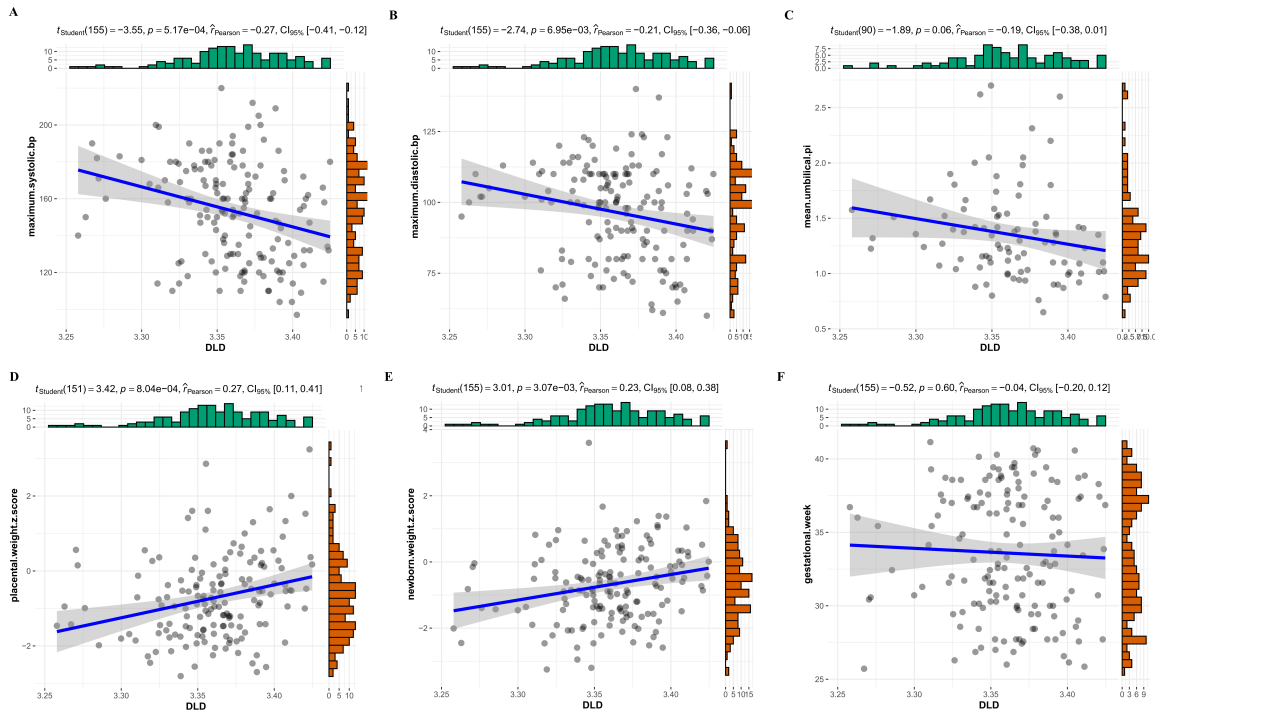


**Figure S3 Linear regression model. (A-F).** Linear relationship between DLD gene expression and pregnancy systolic blood pressure, diastolic blood pressure, umbilical artery blood flow ratio, placental weight percentile, fetal weight percentile.


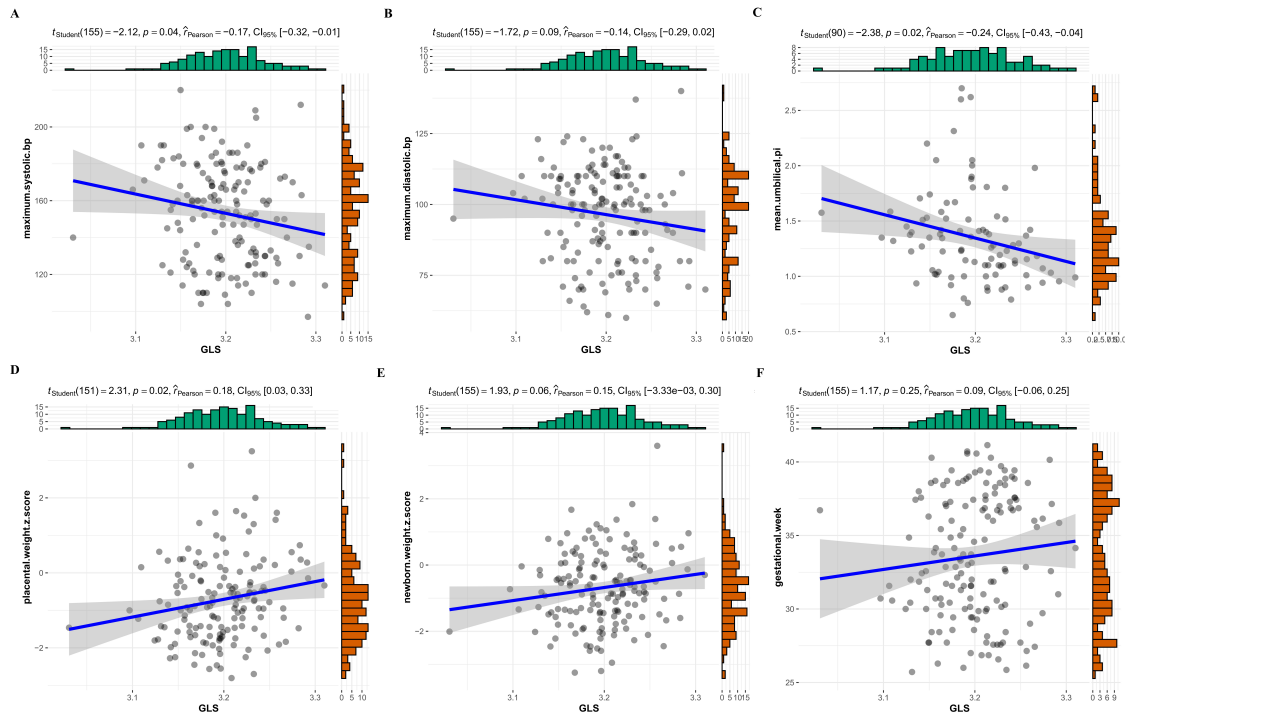


**Figure S4 Linear regression model. (A-F).** Linear relationship between GLS gene expression and pregnancy systolic blood pressure, diastolic blood pressure, umbilical artery blood flow ratio, placental weight percentile, fetal weight percentile.
